# Guidance on the Surgical Management of Rectal Cancer: An Umbrella Review

**DOI:** 10.3390/life15060955

**Published:** 2025-06-13

**Authors:** Ionut Negoi

**Affiliations:** 1 Department of General Surgery, Faculty of Medicine, Carol Davila University of Medicine and Pharmacy Bucharest, 050474 Bucharest, Romania; ionut.negoi@umfcd.ro; 2 Clinical Emergency Hospital of Bucharest, 014461 Bucharest, Romania

**Keywords:** rectal cancer, guidance, surgical management, multimodal approach, high-volume centers, minimally invasive techniques, robotic surgery, transanal TME

## Abstract

This umbrella review synthesizes international guidelines on the surgical management of rectal cancer to provide unified recommendations tailored to local healthcare organizations. This review emphasizes the importance of surgical centralization in high-volume centers, which maximizes outcomes, reduces morbidity, and increases survival rates. Minimally invasive approaches, such as laparoscopy and robotic surgery, are highlighted for their perioperative benefits, although careful patient selection and surgical expertise are required. Mechanical bowel preparation combined with oral antibiotics is recommended to effectively reduce complications, including surgical site infections and anastomotic leakage. Enhanced Recovery After Surgery protocols have been shown to significantly improve postoperative recovery and reduce hospital stay duration. Comprehensive perioperative care, including venous thromboembolism prophylaxis and infection control, is essential for optimal patient outcomes. This review underscores the need for structured training, certification, and regular audits for advanced techniques such as robotic surgery and transanal total mesorectal excision. Implementation of a national database is recommended to support ongoing improvements in rectal cancer surgery. This review centralizes evidence-based recommendations to guide surgical decision-making and harmonize the multidisciplinary care for patients with rectal cancer.

## 1. Introduction

Rectal cancer is an ongoing public health challenge with increasing incidence rates, particularly in the younger population [1]. The complexity of treatment protocols and the potential for severe health consequences stemming from both the disease and its interventions make this malignancy particularly challenging to treat. The multidisciplinary nature of rectal cancer management, involving surgeons, imagists, gastroenterologists, oncologists, radiologists, and pathologists, underscores the need for coordinated care [2,3,4,5,6].

Recent epidemiological trends have shown a worrying increase in rectal cancer cases among individuals aged < 50 years, a demographic traditionally considered low risk [7]. This shift necessitates a re-evaluation of screening guidelines and treatment approaches. The complexity of rectal cancer treatment, which may include surgery, radiation, and chemotherapy, can lead to considerable patient morbidity, including bowel dysfunction, sexual and urinary problems, and the potential need for a permanent ostomy.

Moreover, the intricacy of surgical techniques, such as open, laparoscopic, robotic-assisted, and transanal approaches, demands high levels of expertise to ensure optimal oncological results while maximizing quality of life. The multifaceted nature of rectal cancer care highlights the importance of specialized centers and standardized treatment protocols for improving patient outcomes and reducing treatment-associated complications [8].

The aim of this study is to review the existing guidelines, to unify and standardize the approach to rectal cancer management while accounting for local healthcare resources. By consolidating diverse guidelines, a relevant framework that addresses the specific needs and resources of the healthcare system can be developed. A unified approach may facilitate consistent, high-quality care across different institutions, potentially improving patient outcomes. This guidance does not encompass all available evidence and treatment alternatives for patients with rectal cancer, nor does it address all clinical particularities, primary and secondary diagnoses, or clinical complexities of specific patients, and should be adapted to individual patient preferences. The present material aligns with the current medical literature, acknowledging its recognized heterogeneity, and may be used to guide different clinical scenarios without representing a definitive statement of the standard of care [9]. The author of this paper and the other two emerged from the same scientific project does not support or recommend any external services, treatments, equipment, or medications. He accepts no responsibility or liability for any negative consequences that may result from or be associated with the use of this information [9].

## 2. Methods

An umbrella review of rectal cancer guidelines was performed, including those of the European Society of Coloproctology (ESCP), the Association of Coloproctology of Great Britain and Ireland (ACPGBI), the National Institute for Health and Care Excellence (NICE), the European Society for Medical Oncology (ESMO), the American Society of Colon and Rectal Surgeons (ASCRS), the National Comprehensive Cancer Network (NCCN), the Japanese Society guidelines (JSCCR), and the Chinese national guidelines [10]. An examination of the current global guidelines was performed, along with a systematic exploration of the PubMed/MEDLINE, Web of Science, and Semantic Scholar databases to provide additional corroborating evidence. The guidance is structured into three sections: the first encompasses surgical anatomy, adjuvant therapy, follow-up, and surveillance [6], the second addresses neoadjuvant therapy, while the current article focuses on surgical management.

This study is compliant with the Preferred Reporting Items for Systematic Reviews and Meta-Analysis (PRISMA) 2020 statement [11]. The review protocol was prospectively registered in the Open Science Framework database (OSF—osf.io/e3dy7).

### 2.1. Eligibility Criteria

The inclusion criteria were as follows: published clinical practice guidelines, guidance documents, or consensus statements focusing on rectal cancer surgical techniques issued by major international or national organizations. High-level evidence from primary research, specifically randomized controlled trials (RCTs) and meta-analyses pertaining to rectal cancer surgery, was also included, provided they were published in high-impact (Q1 and Q2) peer-reviewed journals. Exclusion criteria: Articles that did not present formal guidance or high-level evidence were excluded. This included single-case reports or small case series and any sources not indexed in recognized databases (e.g., informal publications or gray literature). Studies focusing only on the non-surgical aspects of rectal cancer management (such as purely medical oncology or radiation therapy guidelines without surgical techniques) were also excluded.

### 2.2. Information Sources and Search Strategy

A structured literature search was performed to retrieve relevant guidelines and studies with high levels of evidence. PubMed/MEDLINE, Web of Science, and Semantic Scholar were searched for eligible records published from 1 January 2010, to 31 December 2024 (with an update in early 2025 to capture any newly published materials) (Table 1). Included studies were cross-referenced for any supplementary relevant publications.

### 2.3. Quality Appraisal

No formal risk of bias assessment or quality scoring was performed for the included studies and guidelines. The focus of this umbrella review was to collate and summarize recommendations and findings from high-level evidence sources rather than to grade the methodology of each source. All included guidelines were assumed to be of high quality, as they were produced by recognized professional organizations, while the RCTs and meta-analyses were retrieved from Q1 and Q2 journals.

### 2.4. Data Synthesis

Qualitative synthesis of the collected evidence was performed, as no quantitative meta-analysis was appropriate given the nature of the data. The findings are presented descriptively, grouping consistent themes and recommendations across different guidelines and comparing areas of agreement or divergence between them. Supportive evidence from RCTs and meta-analyses was integrated to reinforce or contrast with these recommendations. For the present review, the following software was utilized: Microsoft Excel and Paperpile LLC 2025. The artificial intelligence software Microsoft Office, Web of Science, Perplexity AI and Chat GPT was used for proofreading of the manuscript, and Litmaps for cross-referencing [12].

## 3. Results

**Figure 1:**
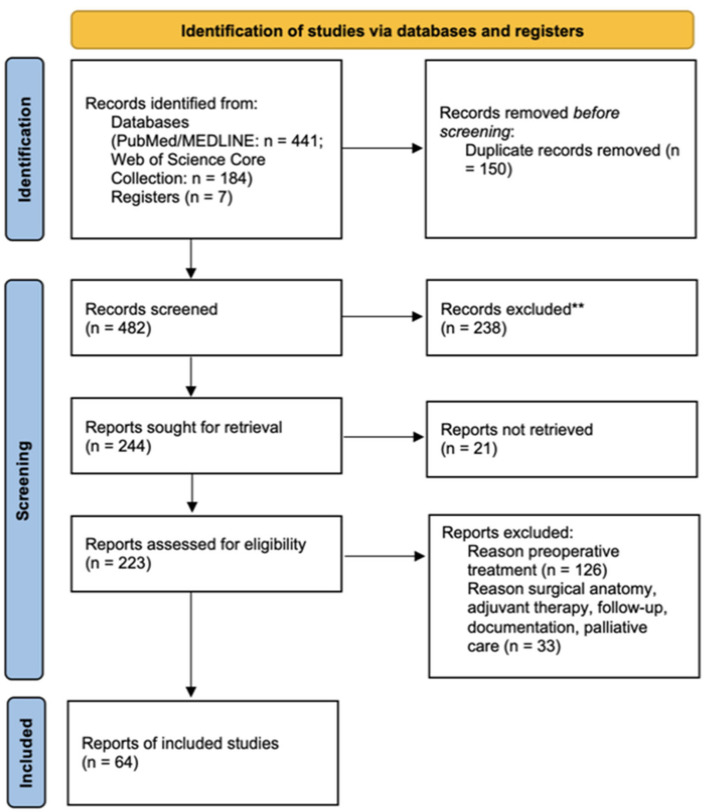
PRISMA 2020 flow diagram for the review of the literature [11].

Of the 482 papers retrieved, 64 scientific articles were included in the qualitative synthesis (Figure 1).

### 3.1. Preoperative Optimization and Surgical Planning

#### 3.1.1. Mechanical Bowel Preparation and Oral Antibiotics to Reduce Surgical Complications

Mechanical bowel preparation (MBP) and oral antibiotics (OA) offer clinical benefits when radical proctectomy with anastomosis is planned [13]. To mitigate the risk of surgical site infections, preventive strategies should be employed, including the delivery of a single dose of broad-spectrum antibiotics before the initiation of the surgical procedure [13].

Recent research has indicated that the administration of oral antibiotics prior to surgical procedures can significantly reduce the incidence of surgical site infections (SSIs) following rectal surgery. This benefit was observed when oral antibiotics were used in conjunction with intravenous antibiotics and mechanical bowel preparation. A randomized controlled trial evaluated two treatment protocols: one cohort received oral antibiotics (erythromycin 500 mg and metronidazole 500 mg, administered one day before surgery) in addition to intravenous antibiotics (cephalosporin), while the other cohort received only intravenous antibiotic prophylaxis. The findings for rectal resections revealed SSI rates of 3.5% and 22% (*p* = 0.002) in the combined and intravenous-only groups, respectively [14]. The disparity was apparent in both superficial surgical site infections (SSIs) (0% versus 8.5%, *p* = 0.03) and deep/organ space SSIs (3.5% versus 15.3%) [14].

A study by Kiran et al. examining data from 8442 patients undergoing elective colorectal surgeries via the National Surgical Quality Improvement Program resolved a decades-long debate. In multivariable analysis, MBP + OA correlated with fewer anastomotic leaks (OR 0.57 95%CI 0.35–0.94), postoperative ileus (OR 0.71, 95%CI 0.56–0.90), and SSIs (OR 0.40, 95%CI 0.31–0.53) [15].

Another RCT conducted in China demonstrated that the addition of OA to MBP and systemic antibiotic prophylaxis significantly reduced SSIs (8.42% versus 27.3%, *p* = 0.004) and readmission rates in clean contaminated, contaminated, and dirty colorectal resections [16]. Multivariable analysis indicated that the inclusion of OA decreased the incidence of SSIs (OR 0.20, 95%CI 0.06–0.6, *p* = 0.005) [16].

A meta-analysis synthesizing data from three randomized controlled trials involving 1136 participants demonstrated significant reductions in various types of surgical site infections (SSIs) when oral fluoroquinolone antibiotics were administered preoperatively alongside intravenous antibiotic prophylaxis and mechanical bowel preparation. The study identified substantial decreases in organ/space (RR 0.34; 95%CI 0.12–0.90), deep incisional (RR 0.19, 95%CI 0.06–0.65), and superficial (RR 0.38, 95%CI 0.22–0.58) SSIs under these conditions [17].

The administration of oral antibiotics may reduce the incidence of critical postoperative complications in rectal resections, such as anastomotic leakage [16,17]. Johnson et al. demonstrated that the adding oral fluoroquinolones to intravenous antibiotic prophylaxis and MBP decreased the anastomotic leakage (RR 0.22, 95%CI 0.06–0.87, I^2^ = 0%) [17].

#### 3.1.2. Enhanced Recovery After Surgery (ERAS) Protocols

The management of patients undergoing planned surgical resection throughout the perioperative period should be guided by the ERAS protocol.

The ERAS protocol is an effective, feasible and safe approach for rectal surgery. It offers a structured methodology that reduces physiological stress and facilitates recovery [18,19]. ERAS protocols offer several benefits to patients undergoing rectal surgery, including reduced complications, shorter hospital stay, and improved recovery outcomes. Between 2009 and 2015, a significant reduction in the mean hospital stay was observed, decreasing from 12.3 to 5.7 days (*p* < 0.001) [20], with conventional care showing a duration of 9 versus 6 days [21]. This reduction was consistent with the results of various studies [22,23]. The implementation of ERAS protocols has been demonstrated to significantly reduce the incidence of surgical site infections from 24% to 5% and decrease the occurrence of postoperative ileus from 39% to 6% [20]. The overall morbidity rates were significantly lower in the ERAS groups than in those receiving traditional care, with rates of 14.8% versus 33.6% (*p* < 0.01) [21]. ERAS protocols accelerate the achievement of recovery benchmarks, including quicker intervals to oral feeding, improved mobility, and reduced time to the first flatus passage. This is achieved through preoperative carbohydrate loading and early initiation of postoperative nutrition [22]. While some hospitals report an 84.2% adherence rate, along with a 25.2% reduction in hospital-acquired infections and an 18.2% decrease in SSIs [24], compliance with the ERAS protocol remains challenging, and the relative benefits of all its components are still unclear [25].

#### 3.1.3. Venous Thromboembolism (VTE) Prophylaxis Strategies and Their Duration

A comprehensive strategy is essential for preventing VTE in patients undergoing surgical procedures. This strategy should include the use of perioperative low-molecular-weight heparin (LMWH), graduated compression stockings, and intraoperative intermittent pneumatic compression devices. Furthermore, it is recommended that these prophylactic measures be continued for 28 days postoperatively [13].

Although relatively low, the incidence of VTE following rectal surgery persists at a higher rate compared to those receiving other surgeries, with an overall prevalence of 1.25% (95%CI 0.86 to 1.63) [26,27]. The incidence of deep vein thrombosis (DVT) was 0.68% (95%CI, 0.48–0.89), while pulmonary embolism (PE) was noted in 0.57% (95%CI, 0.47–0.68) of patients undergoing rectal resections [26]. The incidence of VTE after laparoscopic resection was 0.58%, whereas after open resection was 2.22% [26].

A systematic review and meta-analysis of four RCTs evaluated the impact of extended prophylaxis (4 weeks of LMWH) compared to the conventional regimen (1 week of LMWH) following major abdominal and pelvic surgery [28]. To prevent one instance of VTE, overall, and proximal DVT, it was necessary to treat 14, 14, and 44 patients, respectively. The authors concluded that extended prophylaxis is associated with a reduction in asymptomatic VTE and should be considered in high-risk patients [28].

### 3.2. Surgical Techniques and Approaches

Radical surgical interventions for rectal cancer include low anterior resection, ultralow anterior resection with coloanal anastomosis, and abdominoperineal resection, all of which also involve total mesorectal excision (TME) [29]. At the beginning of the surgery, comprehensive surgical exploration should be conducted following the abdominal entry [30].

#### 3.2.1. Open and Minimally Invasive Approaches

Open surgery is recommended for patients with locally advanced tumors, those with acute complications, those requiring multivisceral resection, those with severe adhesions or distorted anatomy, and when minimally invasive approaches are contraindicated [29].

A minimally invasive approach, laparoscopic or robot-assisted, can be considered by experienced surgeons with technical expertise and is indicated for patients without locally advanced tumors (considering tumor location and progression) and without threatened or high-risk circumferential margins [29,31]. Patient factors (e.g., obesity and previous abdominal surgeries) should also be considered [31].

Transanal TME (TaTME) may be considered for low rectal cancers that pose difficulties with conventional methods, especially in patients with a narrow pelvis or obesity. However, this technique should only be employed in research settings and under close professional supervision. Evidence of its efficacy is adequate, but evidence of its safety is inconsistent, including associated injuries and intraoperative malignant dissemination [32].

For middle and lower rectal cancers, transanal TME demonstrates overall complication rates and functional outcomes comparable to those of the laparoscopic TME and robotic TME approaches [33].

#### 3.2.2. Vascular Division

For rectal cancer resection, it is typically appropriate to perform vascular division of the inferior mesenteric artery (IMA) inferior to the left colic artery branching off. Regardless of whether the IMA is transected above or below the point where the left colic artery branches off, it is essential to remove the central lymph nodes situated near the IMA origin.

The debate between high versus low tie ligation of the IMA during rectal resection remains critical in colorectal surgery. After reviewing 29 studies, Brillantino et al. presented a mean length of the IMA from the take-off to its first branch of 39.3 mm, around which the JSCCR defined lymph node station No. 253 [34]. High ligation, performed at the IMA origin, aims to enhance lymph node retrieval and staging accuracy. This approach has been associated with a higher likelihood of lymphadenectomy, potentially allowing a clearer oncological margin due to better regional lymphatic drainage; however, it does not consistently correlate with improved patient survival outcomes [35,36,37]. Conversely, low ligation, achieved below the left colic artery, often results in fewer postoperative complications, particularly anastomotic leakage and bowel ischemia [38,39]. Studies have indicated that the preservation of the left colic artery can offer better perfusion to the remaining bowel, thereby minimizing ischemic risks and enhancing recovery [40,41]. Moreover, low tie ligation may contribute to improved functional outcomes, including sexual and urinary function post-surgery, making it a compelling option for surgeons prioritizing quality of life after rectal cancer resection [40,42].

Thus, while high ligation facilitates lymph node dissection, low ligation presents tangible benefits concerning postoperative recovery and complications, highlighting the need for individualized surgical planning based on patient-specific factors [38,39,43]. This should be a nuanced decision with an individualized approach, considering the patient’s anatomy, preoperative imaging, intraoperative perfusion assessment, and surgical expertise to integrate the potential impact on anastomotic leakage, genitourinary function, and oncological outcomes [34].

#### 3.2.3. Mesorectal Excision

TME involves sharp dissection along the mesorectal fascia to remove the rectum and its surrounding mesorectum intact, preserving the autonomic nerves to minimize functional deficits. Partial mesorectal excision is suitable for upper rectal tumors, with the mesorectum transected no less than 5 cm below the inferior tumor margin. For cancers in the middle and lower rectum, total mesorectal excision is required and is performed as part of either low anterior or abdominoperineal resection. A distal safety margin of 2 cm is typically recommended for the rectal wall. However, for tumors situated below the inferior mesorectal margin, a margin of 1 cm may be considered acceptable [30]. In the context of T4 tumors, surgical intervention involving multiple organs is necessary to ensure complete tumor removal and achieve an R0 resection. Specimen extraction should be performed via a protected incision to reduce wound contamination, with careful attention to cancer recurrence, cosmetic outcomes, and infection prevention.

### 3.3. Surgical Expertise, Training, and Institutional Volume

The operating surgeon should have experience in rectal cancer surgery, including TME. In cases with threatened CRM or positive lateral lymph nodes, the operating surgeon should have experience in “beyond TME” resections, and a multidisciplinary team should be available [29]. Institutions, divisions, and surgeons implementing new minimally invasive procedures should participate in a structured training, mentoring, and evaluation program supervised by an institutional body.

Evidence suggests that surgeons who perform a higher number of rectal cancer surgeries annually tend to achieve better surgical outcomes [44]. This highlights the importance of experience and specialization in improving patient care in rectal cancer surgery. High-volume centers and specialized training programs are essential for optimizing surgical success and patient survival [45].

A study examining surgical procedures in New York from 2000 to 2013 found a significant increase in operations performed by surgeons with high cumulative and high annual volumes (HC-HAV). The percentage of such procedures rose from 38.3% to 58.4% during this period (*p* < 0.01) [44]. Surgeons classified as having HC-HAV demonstrated a notably reduced incidence of surgical complications (OR 0.71, 95%CI 0.60–0.83, *p* < 0.05). However, there were no differences in terms of anastomotic leakage, non-routine discharge, or readmission rates [44].

A meta-analysis of 21 high-quality studies demonstrated a notable protective relationship between hospital volume, rather than surgeon volume, and the outcomes of rectal cancer surgery [45]. Hospitals with high patient volumes demonstrated reduced postoperative complications (OR 0.80, 95%CI 0.70–0.93), mortality (OR 0.62, 95%CI 0.43–0.88), definitive colostomies (OR = 0.51, 95%CI, 0.29–0.92), and improved OS rates (OR = 0.99, 95%CI, 0.98–1.00) [45].

In Germany, 53% of colorectal cancers are treated at certified centers (CRC-CC) [46]. Compared with conventional care, CRC-CC demonstrated a significantly higher rate of laparoscopic procedures for rectal cancer (55% vs. 5%, *p* < 0.001), extended operation duration (192 vs. 161 min, *p* < 0.001), and reduced incidence of anastomotic leakage (0% vs. 2%, *p* = 0.036). However, the overall morbidity rates were similar (38% vs. 32%, *p* = 0.174). Notable improvements were observed in the quality of histopathological examinations, overall survival, and stage IV disease outcomes (*p* = 0.041). For stage III disease, the difference in DFS did not reach statistical significance (*p* = 0.050) [46].

A Cochrane systematic review and meta-analysis demonstrated a clear correlation between surgical volume and outcomes in colorectal cancer procedures, considering both hospital and surgeon caseloads as well as specialization [47]. A significant association was observed for patients with rectal cancer between high-patient-volume hospitals and improved 5-year survival rates (HR 0.85, 95%CI 0.77–0.93). However, this association was not evident for postoperative deaths (OR 0.97, 95%CI 0.70–1.33). The number of patients per surgeon did not show a correlation with 5-year survival (HR 0.99, 95%CI 0.86–1.14) or operative mortality (OR 0.86, 95%CI 0.62–1.19). Both high-volume hospitals and surgeons exhibited significantly lower rates of definitive stoma (OR = 0.64, OR = 0.75, respectively) and abdominoperineal resection (APR) (OR = 0.55, OR = 0.70, respectively) [47].

An analysis of 13,858 patients with rectal cancer treated in 166 UK hospitals between 2015 and 2019 showed that 45% of surgeons performed less than five resections per year [48]. Nevertheless, no differences were observed in relation to hospital and surgeon volume for mortality within 90 days, unplanned readmissions within 30 days, presence of stoma at 18 months, positive circumferential resection margin, or all-cause mortality at 2 years [48]. The authors concluded that centralizing rectal cancer surgery to increase operative volume had no impact on the results, and multiple interventions were necessary across the multidisciplinary pathway [48].

### 3.4. Technical Aspects and Intraoperative Decision-Making in Low Rectal Cancers

Lower rectal cancer should be approached based on appropriate MRI evaluations [49]. Patients with low rectal cancer, above the anal sphincter, without threatening (>1 mm) the mesorectal fascia and puborectalis muscle, and with a clear mesorectal dissection plane, may be approached by TME and coloanal anastomosis or APR (conventional or intersphincteric). On the other hand, for low rectal cancers (i) above the sphincter and threatening the mesorectal fascia or the puborectalis muscle or (ii) at the level of the sphincter and invading the intersphincteric plane, extralevator abdominoperineal resection (ELAPE) should be performed. ELAPE is associated with a lower intraoperative perforation rate [50].

A sphincter-preserving procedure should be performed only if negative distal and circumferential margins can be obtained, and if the postoperative anal function is reasonably expected to be satisfactory [31]. Nerve-sparing techniques should be employed to preserve the hypogastric and pelvic splanchnic nerves during TME to ensure optimal urinary and sexual function.

Intersphincteric resection may be used for low rectal tumors in patients with good anal sphincter tonus to obtain an adequate, two-centimeter, distal margin. This procedure involves resection of the internal anal sphincter. A clear CRM necessitates an intersphincteric plane free of tumor infiltration, with no involvement of the external anal sphincter or puborectalis muscle. However, this procedure is contraindicated for poorly differentiated tumors [31].

Following transection of the distal rectum, the rectal stump may be cleansed to decrease the number of malignant cells shed within the lumen [13,30]. Following low anterior resection, surgeons may opt to perform side-to-end anastomosis or a colonic J-pouch. These techniques are considered potential methods for enhancing the functional outcomes of the procedure [30]. Following the completion of colorectal anastomosis, its integrity should be assessed [30]. For low rectal anastomoses, it is advisable to consider creating a diverting stoma, with loop ileostomy being the favored option [13,30].

### 3.5. Management of Lateral Pelvic Lymph Nodes

Selective lateral lymph node dissection is recommended, particularly in patients whose lateral nodes show radiological evidence of involvement and have not responded significantly to preoperative treatment.

According to the Western centers’ approach, local control of lateral lymph nodes that may harbor tumor cells is achieved by total neoadjuvant therapy or neoadjuvant radiochemotherapy, followed by complete mesorectal excision. However, enlarged lymph nodes that are evident on pretherapeutic MRI evaluation and persist after neoadjuvant therapy, if located outside the mesorectum, will be identified and surgically resected, usually by a complete lateral lymphadenectomy.

An analysis of recurrence patterns following rectal surgery in the Dutch TME study, which included 713 patients receiving radiotherapy in conjunction with TME and 704 patients undergoing TME alone, identified 114 local recurrences (LR). The 5-year LR rates were 4.6% and 11.0% in the RT + TME and TME-only groups, respectively. Presacral recurrence was observed in both groups. Radiotherapy reduced the anastomotic LR rate, except in cases with a distal margin of less than 5 mm. Abdominoperineal resection was associated with presacral local recurrence. Lateral local recurrences account for 20% of all local recurrences [51].

According to Eastern countries’ concepts [31], lymphadenectomy of the pelvic lymph nodes (internal iliac, obturator) is indicated in cancers whose lower margin is located in the extraperitoneal rectum, and the tumor invades outside the muscularis propria (T3,T4) [52]. Lateral pelvic dissection, routinely performed in Eastern countries, is accompanied by technical and anatomical challenges, and the additional morbidity associated with this procedure has led to its limited implementation in Western countries. Surgical access for lateral lymphadenectomy must follow specific anatomical landmarks [53]. The peritoneum should be opened along the ureters, with the medial limits being the ureters and neurovascular structures, hypogastric nerves, and lower hypogastric plexuses. The lateral border of the dissection is the external iliac vein, with communication between the inguinal and iliac nodes being interrupted and then entering the obturator fossa. A critical step is the identification of the obturator nerve through blunt dissection of the fat at this level. Dissection was completed by separating the specimen from the internal iliac vessels and emerging branches at this level [53].

In a study examining advanced lower rectal cancer, Yokoyama et al. assessed the impact of lateral lymphadenectomy on patient survival [54]. Their study included 131 consecutive patients who underwent radical surgical interventions. Radical resection comprises two principal components: complete mesorectal excision and extended lateral lymphadenectomy [54]. Lateral node invasion was observed in 19.1% of patients. Multivariate analysis identified that both the number of invaded nodes (>1) and their anatomical location (common or external iliac) served as significant predictors of recurrence and survival outcomes. Notably, patients with a single invaded node located in the internal iliac or obturator region showed improved survival rates [54].

Schaap et al. [55] investigated the impact of obturator and internal iliac nodes on cancer outcomes. Their findings indicated that enlarged internal iliac nodes, characterized by a short MRI axis greater than 7 mm before treatment and 4 mm after radiochemotherapy (HR = 5.74, 2.98–11.05 vs. HR = 1.4, 0.19–10.20, *p* < 0.001, compared to nodes smaller than 4 mm post-therapy), were associated with an increased rate of local lateral recurrence. Furthermore, enlarged obturator nodes (short axis > 6 mm following radiochemotherapy) were linked to more advanced disease stages, the presence of distant metastases, and reduced cancer-specific survival in patients who did not undergo lateral lymphadenectomy. Lateral lymphadenectomy did not correlate with improved survival, with only cT (HR = 2.22, 1.07–4.64) and resection margin invasion (HR = 2.95, 1.11–7.37) associated with the development of metastatic disease [55].

A study involving 1216 patients with advanced lower rectal cancer (cT3-T4) from both Western and Eastern medical centers demonstrated that lymph nodes with a short axis exceeding 7 mm on pretreatment MRI were correlated with a 19.5% local recurrence rate following neoadjuvant radiochemotherapy and total mesorectal excision [56]. The recurrence rate decreased to 5.7% (*p* = 0.042) after lateral lymphadenectomy [56].

Following radiochemotherapy (RCT), no local recurrence was observed when lymph nodes initially measuring > 7 mm in the short axis were reduced to less than 4 mm in the internal iliac region and <6 mm in the obturator area [57].

Kawai et al. [58] studied 279 rectal cancer patients receiving neoadjuvant chemoradiotherapy followed by resection, between 2007 and 2018. The study identified a 9.3% incidence of lateral lymph node metastases. Notably, 40.4% of patients with lateral lymph node metastases remained free of recurrence during a surveillance period exceeding five years. The study indicated that a pre-chemoradiotherapy lateral lymph node size of ≥8 mm might be an optimal threshold for performing lateral lymphadenectomy, with a sensitivity of 92.3% and specificity of 78.7%. This criterion facilitates the avoidance of lateral lymph node dissection in 72% of cases while achieving curative dissection in >30% of patients with lateral lymph node metastases [58].

A non-inferiority randomized controlled trial conducted by Fujita et al. investigated 701 patients with stage II/III lower rectal cancer, specifically those without enlarged lateral lymph nodes. The study participants were randomly allocated to undergo either TME alone or TME in conjunction with lateral lymphadenectomy (TME + LLND) [59]. The TME + LLND cohort presented 5-year OS and 5-year local recurrence-free survival rates of 92.6% and 90.2%, respectively. Conversely, the TME cohort demonstrated rates of 87.7% and 82.4% for the same metrics, respectively. Local recurrence was observed in 7.4% of cases in the TME + LLND group and 12% in the TME group (*p* = 0.024). The researchers concluded that TME alone did not demonstrate non-inferiority to TME + LLND, with the latter approach yielding a lower rate of local recurrence [59]. Long-term monitoring of this patient group revealed that TME could not be proven to be non-inferior to TME combined with LLND [60]. Subgroup analysis demonstrated improved relapse-free survival in stage III disease (HR = 1.49, 1.02–2.17) [60].

A meta-analysis of 18 studies with a total of 6133 patients demonstrated no statistically significant differences in 5-year OS (OR 1.01, 0.78–1.30), 5-year disease-free survival (OR 1.07, 0.86–1.32), or local recurrence (OR 1.01, 0.72–1.42). Nevertheless, the integration of TME and LLND was associated with prolonged operative duration (mean difference: 116.02, 89.20–142.83), an elevated incidence of postoperative complications (OR 1.59, 1.14–2.24), urinary dysfunction (OR 6.6, 3.31–13.39), and sexual dysfunction (OR 9.67, 2.38–39.26). These results were consistent across subgroup analyses of both randomized and observational trials, irrespective of the administration of neoadjuvant chemoradiotherapy [61].

## 4. Discussions

### 4.1. Short-Term Outcomes of Surgical Approaches

A meta-analysis of high-quality evidence revealed no significant differences between laparoscopic and open surgical approaches in terms of blood loss, postoperative outcomes (such as length of hospital stay following surgery), and postoperative complications [62]. However, the analysis indicated that laparoscopic procedures required an average of 41.99 min longer to complete than open surgery, a difference that was statistically significant (*p* < 0.00001) [62].

A randomized controlled trial conducted in China involved 1070 patients who were assigned to undergo either laparoscopic or open rectal resection [63]. Both cohorts showed comparable pathological outcomes, including complete mesorectal excision rates, negative margins, and lymph node retrieval rates. The laparoscopic group demonstrated advantages in recovery, with shorter times to first flatus (40.4 versus 44.8 h) and defecation (61.2 versus 66.3 h), reduced analgesic use (45 vs. 48 h), and shorter hospital stays (8 vs. 9 days). The laparoscopic approach showed fewer postoperative complications (13.0%) than the alternative method (17.2%), although the difference was not statistically significant (*p* = 0.065). The conversion rate was 2.5%, with no 30-day mortality in either group. When performed by skilled surgeons, laparoscopic procedures achieve outcomes comparable to traditional open surgery in patients with low rectal cancer while offering improved recovery [63].

### 4.2. Pathological Outcomes of Surgical Approaches

A meta-analysis comparing laparoscopic versus open mesorectal excision showed higher positive circumferential resection margins in laparoscopic procedures (8.5%, 49/574 patients) versus open resections (5.4%, 30/557 patients), with a relative risk of 1.55 (95%CI 0.99 to 2.41, *p* = 0.05). No heterogeneity was found among the studies (I2 = 0%). Incorrect mesorectal excision occurred in 11% of laparoscopic and 8.4% of open surgeries (*p* = 0.18). The number of lymph nodes excised and the distance to the distal margin showed no significant differences between the approaches [62].

A separate meta-analysis by Martinez-Perez, published in JAMA Surgery, analyzed 14 high-quality randomized controlled trials [64]. The findings indicated that patients who underwent laparoscopic resection faced a significantly higher risk of non-complete mesorectal excision (either incomplete or near-complete) than those who underwent open resection [64]. In the laparoscopic group, non-complete mesorectal excision was 13.2% (179/1354), compared to 10.4% (104/998) in open surgeries (RR = 1.31, 95%CI 1.05–1.64; *p* = 0.02; I2 = 0%). Among 2989 patients, positive circumferential resection margins were 7.9% in laparoscopic versus 7.1% in open procedures (RR = 1.17, 95%CI 0.89–1.53, *p* = 0.26, I2 = 0%). No significant differences were found between the groups in excised lymph nodes (MD 0.05; 95%CI −0.77–0.86, *p* = 0.91), distances to distal (MD 0.01 cm, 95%CI −0.12–0.15 cm, *p* = 0.87), radial margins (MD −0.67 mm, 95%CI −2.16–0.83 mm, *p* = 0.38), or distal margin invasion (RR 1.12; 95%CI 0.34–3.67, *p* = 0.86) [64].

In 2019, a non-inferiority meta-analysis evaluating the quality of surgical resection outcomes was published in the Annals of Surgery, including data from 14 RCTs [65]. The non-inferiority margins (Δ_NI_) were established by consensus among 58 international experts. The laparoscopic approach showed non-inferiority to open resection for positive CRM rate (RD 0.79%, 90%CI −0.46–2.04, Δ_NI_ 2.33%, PNI 0.026), invaded distal resection margin (RD 0.15%, 90% CI −0.58–0.87, Δ_NI_ 1.28, PNI 0.005), and incomplete mesorectal excision plane (RD 1.16%, 90%CI −0.27–2.59, Δ_NI_ 2.85%, PNI 0.025). Using expert-generated Δ_NI_, the “successful resection” rate was indeterminate (RD 6.16%, 90% CI 2.30–10.02) [65].

A network meta-analysis of histopathological outcomes from rectal cancer resection techniques was conducted by the Association Française de Chirurgie, comparing open, laparoscopic, robotic, and transanal approaches [66]. Analysis of 27 RCTs with 8696 patients showed that transanal (OR 0.60, 95%CI 0.33–0.92, *p* = 0.02) and robotic (OR 0.68, 95%CI 0.46–0.94, *p* = 0.02) rectal resection techniques had a reduced incidence of non-complete mesorectal excisions compared to laparoscopic procedures. Robotic and transanal methods showed comparable outcomes to open surgery (OR 0.79, 95%CI 0.39–1.36, *p* = 0.36; OR 0.89, 95%CI 0.52–1.43, *p* = 0.61). Surface under cumulative ranking (SUCRA) values for optimal treatment outcomes were as follows: transanal (0.918), robotic (0.617), open (0.412), and laparoscopic (0.054).

In the SUCRA rankings for negative circumferential resection margins, the transanal approach scored the highest (0.918), followed by robotic (0.617), open (0.412), and laparoscopic (0.054) methods. The transanal technique showed an advantage over the laparoscopic approach for positive CRM rate (OR 0.36, 95%CI 0.13–0.91, *p* = 0.02). For negative distal margins, the SUCRA rankings were as follows: open (0.8), robotic (0.557), transanal (0.548), and laparoscopy (0.095), with no significant differences among the approaches. The robotic technique yielded more lymph nodes than laparoscopy (MD 1.24, 95%CI 0.10–2.52, *p* = 0.03), while the transanal method yielded fewer nodes than laparoscopy (MD −1.9, 95%CI −3.77 to −0.18, *p* = 0.03), open surgery (MD −2.01, 95%CI −4.16 to −0.09, *p* = 0.04), and robotic procedures (MD −3.15, 95%CI −5.46 to −1.11, *p* = 0.005) [66].

A network meta-analysis of 32 RCTs compared the open, laparoscopic, robotic, and transanal approaches for TME [67]. Robotic TME showed better distal resection margins and shorter hospital stays, with no significant differences in other recovery and oncological outcomes [67].

The results of the COLOR III trial [68], a multicenter international superiority RCT comparing transanal and laparoscopic TME, are awaited. For this study, a quality assurance system (SQA) was developed, incorporating a competency assessment tool comprising nine steps divided into four stages [69]. A similar RCT conducted by the Chinese Transanal Endoscopic Surgery Collaborative (CTESC) Group revealed comparable results for a 3-year DFS rate (83.4% vs. 82.8%) and local recurrence rate (3.6% vs. 3.9%) between the laparoscopic (laTME) and transanal (taTME) approaches [70].

### 4.3. Long-Term Oncological Outcomes of Surgical Approaches

A meta-analysis of 12 RTCs (3709 patients) evaluated laparoscopic versus open proctectomy for cancer [71]. The 5-year DFS rates were 72.2% for laparoscopic and 70.1% for open resections, showing no significant difference (*p* = 0.26) [71]. The 5-year OS rates were 76.2% and 72.7%, respectively, with a significant difference (*p* = 0.02) [71]. Analysis of seven RCTs (1767 laparoscopic and 1293 open resections) showed no significant differences in DFS and OS between groups (HR 0.91, 95%CI 0.78–1.06, *p* = 0.241; HR 0.86, 95%CI 0.73–1.02, *p* = 0.09) [72]. For stage II and III cancers, laparoscopic surgery showed improved DFS (HR = 0.73, 95%CI 0.54–0.98, *p* = 0.036; HR = 0.74, 95%CI 0.55–0.99, *p* = 0.041) [72].

In a meta-analysis of six studies examining quality of life (QoL) in patients with colorectal cancer after laparoscopic procedures, the results showed significant improvement in physical functioning scores (standardized mean difference = 0.45, 95%CI 0.15 to 0.75, *p* = 0.003) [73]. However, no significant differences were found in other domains, such as general health, the Global Quality Scale, QoL index, physical and mental components, social functioning, and vitality. The authors concluded that the advantages of laparoscopy are modest, with no significant differences in long-term QoL [73].

The COREAN study compared the long-term results of open and laparoscopic radical proctectomy after neoadjuvant chemoradiotherapy. At ten-year follow-up, the survival presented no significant differences between the two approaches. Overall survival was 74.1% for open and 76.8% for laparoscopic surgery, while disease-free survival was 59.3% and 64.3%, respectively [74]. The local recurrence rates were 8.9% for open and 3.4% for laparoscopic procedures, without statistical significance [74]. Laparoscopic procedures offer advantages over open surgery, including reduced blood loss, faster intestinal recovery, and lower postoperative pain. Patients who underwent laparoscopic surgery showed better physical performance and lower fatigue three months postoperatively [75]. The study showed that laparoscopic procedures performed by skilled surgeons do not affect long-term survival outcomes, confirming their viability as an alternative to open proctectomy for cancer [74,76]. The generalization of the results from this study was analyzed, considering the low BMI of patients and study conduction in three high-volume, tertiary South Korean centers, with the participation of only highly trained surgeons who entered the study only after standardized evaluation by a central committee [77].

The COLOR II trial comparing laparoscopic to open surgery showed reduced blood loss in the laparoscopic group (median 200 mL versus 400 mL), longer operative times (240 min versus 188 min), and similar morbidity (40% versus 37%) and mortality (1% versus 2%) [78]. The laparoscopic group had a faster return of intestinal function (two days versus three) and shorter hospitalization (eight days versus nine) [78]. Both groups had a 3-year locoregional recurrence rate of 5%. DFS rates were 74.8% for laparoscopic and 70.8% for open surgery, while the overall survival rates were 86.7% and 83.6%, respectively [79].

The ACOSOG Z6051 trial evaluated laparoscopic versus conventional open surgery in patients with stage II/III rectal cancer, examining pathological outcomes and disease-free survival [80]. The 2-year DFS rates were comparable between the laparoscopic and open resection groups (79.5% and 83.2%, respectively), with similar local recurrence rates (laparoscopic: 4.6%; open: 4.5%). The laparoscopic group showed a slightly lower distant recurrence rate than the open surgery group (14.6% vs. 16.7%) [80]. This study did not establish the non-inferiority of laparoscopic surgery. Effective resection, measured by CRM, distal margin, and TME completeness, was achieved in 81.7% of laparoscopic cases and 86.9% of open surgeries [81]. The study showed that laparoscopic procedures did not demonstrate non-inferiority to open surgery for pathological outcomes. Despite comparable survival and recurrence rates, these findings suggest that laparoscopic surgery may not be optimal for rectal cancer resection [82,83,84,85].

The ALaCaRT study compared the success rates of laparoscopic and open surgery cancer resection [86]. The laparoscopic group achieved an 82% successful resection rate versus 89% in open surgery, failing to meet the non-inferiority criteria [85]. Only 9% of the cases required conversion [86]. Circumferential resection margins were clear in 93% of laparoscopic and 97% of open surgeries, while distal margins were clear in 99% of both. Complete total mesorectal excision was achieved in 87% of laparoscopic and 92% of open procedures [86]. Analysis of the patient-reported outcomes at 12 months post-surgery showed that fewer patients in the open surgery group had moderate-to-severe fecal incontinence, sore skin, and urinary symptoms compared to laparoscopy [87]. At 3 months, no significant differences were observed in bowel or urinary symptoms between the groups. Sexual function scores remained comparable between the groups at 3 and 12 months. However, at three months, fewer women in the open surgery group reported moderate-to-severe sexual dissatisfaction than those in the laparoscopic group [87]. The ALaCaRT study did not establish the non-inferiority of laparoscopic procedures relative to traditional open surgery for successful proctectomy. Although both approaches yielded high-quality outcomes, the trial provided insufficient evidence to endorse laparoscopic surgery as a routine practice for rectal cancer resection. Further long-term data on survival and recurrence are required to fully evaluate these findings.

Spinelli et al. analyzed critically the ACOSOG Z6051 and ALaCaRT RCTs, underlining that the methodological design of non-inferiority did not prove the inferiority of laparoscopy, the chosen values of 6% and 8% for the margins were arbitrary, and the primary endpoint was a pathological outcome and not one that evaluates the patient health and quality of life [88]. Acuna et al. analyzed 71 articles that cited the ACOSOG Z6051 and ALaCaRT studies and found that 28.2% of these articles interpreted incorrectly the results of the two RCTs as proving the inferiority of laparoscopy. In total, 23.5% of articles interpreted correct the ambiguous but correct wording from the two RCTs, and stated that both RCTs were inconclusive [89].

In China, the LASRE study analyzed 1039 participants with low rectal cancer, over five years, with 685 and 354 patients in the laparoscopic and open surgery groups, respectively [90]. The cohort included 380 and 659 patients in TNM stage I and II/III, respectively. The five-year DFS rates for the laparoscopic and open patients were 76.4% and 75.6%, while five-year OS rates were 84.6% and 86.6%, respectively [90,91]. The five-year locoregional recurrence rates were 5.5% and 3.1% in the laparoscopic and open groups, respectively. The authors concluded that when performed by experienced surgeons the laparoscopic approach for low rectal tumors is non-inferior to the open surgery in terms of 3-year DFS [91].

A review of 11 studies with 1079 patients compared robot-assisted surgery to laparoscopic procedures for advanced mid-low rectal cancer after neoadjuvant chemoradiotherapy [92]. Robot-assisted procedures showed advantages, including a shorter distance from the anal verge (0.4 cm, *p* = 0.006), higher complete total mesorectal excision rate (1.93 times, *p* = 0.031), fewer open conversions (OR = 0.324, *p* = 0.017), and reduced hospital stay (WMD = −1.127, *p* = 0.019). Despite requiring a longer operation time (54 min more, *p* = 0.002), robot-assisted surgery presents advantages over laparoscopic techniques [92].

An RCT by Park et al. [93] compared laparoscopic and robotic techniques for treating middle and low rectal cancer. The study found no significant difference in the specimen quality between the approaches. The complete total mesorectal excision (TME) rates were 80.7% for robotic and 77.1% for laparoscopic procedures. However, positive circumferential resection margin (CRM) rates were 0% in the robotic group and 6.1% in the laparoscopic group (*p* = 0.031), a statistically significant difference [93].

A multicenter randomized controlled trial in China (REAL study) assessed robotic versus laparoscopic techniques for middle and low rectal cancers [94,95]. The short-term results showed the superior performance of robotic surgery compared to laparoscopy. The robotic approach achieved higher rates of sphincter-preserving operations (83.1% vs. 76.9%, *p* = 0.008) and macroscopically complete resections (95.4% vs. 91.9%, *p* = 0.012) than the open approach. Robotic surgery showed reduced positive CRM (4.0% vs. 7.1%, *p* = 0.023) and higher median number of harvested lymph nodes (15.0 vs. 14.0, *p* = 0.004) [95]. The study reported less blood loss (40.0 mL vs. 50.0 mL, *p* < 0.001) and fewer intraoperative complications (5.4% versus 8.7%, *p* = 0.029). It also showed reduced 30-day Clavien–Dindo grade II complications (16.1% vs. 22.9%, *p* = 0.003) and shorter hospital stay (7 vs. 8 days, *p* < 0.001). The 30-day postoperative mortality remained consistent between the groups at 0.2% [95]. The long-term data encompassed 1171 patients with a median follow-up of 43 months. Patients who underwent robotic surgery exhibited significantly lower 3-year locoregional recurrence rates (1.5% vs. 4.0%, *p* = 0.025) and higher 3-year DFS rates (87.3% vs. 83.6%, *p* = 0.035) than those who underwent laparoscopic surgery. No significant differences were found in the 3-year OS rates. The research found that robotic surgery for patients with middle and low rectal cancer notably decreased the chances of locoregional recurrence and increase DFS when compared to conventional laparoscopic methods [94].

### 4.4. Limitations of the Current Study

The present study has several limitations. The evidence is heterogeneous, reflecting variable quality and divergent recommendations in international guidelines. Many of the included guidelines rely predominantly on low-to moderate-quality evidence, limiting the certainty of some consolidated recommendations. Additionally, guideline applicability across diverse healthcare infrastructures varies significantly, restricting the generalizability of recommendations to resource-limited settings. The review process itself poses limitations, including potential selection bias by inadvertently excluding non-English guidelines or guidelines not listed in major databases. Furthermore, the absence of formal quality assessments using standardized tools may have resulted in the equal weighting of recommendations with varying credibility. These limitations necessitate the cautious application of the recommendations in clinical practice and careful adaptation for policy development, and underscore the need for further validation studies and ongoing guideline harmonization efforts.

## 5. Conclusions

This umbrella review demonstrates that synthesizing international rectal cancer surgery guidelines into a unified framework can elevate the local standard of care by establishing a common language and consistent benchmarks across diverse health systems. A clear convergence of recommendations underscores the necessity of surgical specialization at high-volume centers to achieve optimal outcomes, with evidence showing that patients treated in such settings experience lower complication rates and improved survival. Adopting uniform, high-quality surgical standards worldwide could minimize the variability in patient results. Furthermore, the guidelines uniformly emphasize robust multidisciplinary collaboration, as coordinated expertise among surgeons, oncologists, radiologists, pathologists, and other specialists ensures comprehensive management plans and has been linked to better oncologic outcomes. In essence, harmonizing international recommendations fosters consistency in practice and allows clinicians to speak a common language in rectal cancer surgery, facilitating the global transfer of best practices and improving patient care.

Equally important, our synthesis highlights the value of minimally invasive surgical techniques, particularly laparoscopic and robotic-assisted resections, when applied to appropriately selected patients by skilled surgeons. These approaches confer perioperative benefits without compromising oncologic efficacy, provided that operative expertise and rigorous patient selection criteria are established. However, to maintain safety and achieve excellent outcomes, advanced techniques should be concentrated in the hands of well-trained teams. This calls for continuous professional development and structured training programs, including formal credentialing and regular audits for innovations such as robotic surgery and transanal total mesorectal excision. The establishment of national and international rectal cancer registries and databases is also recommended to enable ongoing audits, benchmarking, and refinement of surgical practices. As new evidence emerges, clinicians and guideline bodies must be prepared to adapt to ensure that standards of care remain dynamic and evidence-based.

## Figures and Tables

**Table 1:** PICO question and search strategy used in PubMed/MEDLINE database.

**Patients**	Malignant tumors located in the rectum.
**Intervention**	A standardized approach, with pre-established clinical pathways to be used as clinical decision-making support by members of the multidisciplinary team (surgeons, imagists, gastroenterologists, medical oncologists, radiation oncologists, pathologists, etc.).
**Comparison**	No structured approach.
**Outcomes**	Oncological, quality of life, and financial costs.
PubMed search strategy combined keywords and indexed terms (MeSH/headings and free text): “rectal cancer”, “surgery”, “surgical technique”, “guideline”, “consensus”, “RCT”, and “meta-analysis”, supplemented by filters for date range (2010–2025) and English language.	

## Data Availability

All data generated or analyzed during this study are included in this article. Further enquiries can be directed to the corresponding author.
